# Characterization of a non-contact imaging scintillator-based dosimetry system for total skin electron therapy

**DOI:** 10.1088/1361-6560/ab1d8a

**Published:** 2019-06-21

**Authors:** Irwin I Tendler, Petr Bruza, Mike Jermyn, Xu Cao, Benjamin B Williams, Lesley A Jarvis, Brian W Pogue, David J Gladstone

**Affiliations:** 1 Thayer School of Engineering, Dartmouth College, Hanover, NH, United States of America; 2 DoseOptics LLC, Lebanon, NH, United States of America; 3 Department of Medicine, Geisel School of Medicine, Dartmouth College, Hanover, NH, United States of America; 4 Norris Cotton Cancer Center, Dartmouth-Hitchcock Medical Center, Lebanon, NH, United States of America

**Keywords:** surface dosimetry, scintillator, optical imaging, optical imaging, non-contact, remote

## Abstract

Surface dosimetry is required for ensuring effective administration of total skin electron therapy (TSET); however, its use is often reduced due to the time consuming and complex nature of acquisition. A new surface dose imaging technique was characterized in this study and found to provide accurate, rapid and remote measurement of surface doses without the need for postexposure processing.

Disc-shaped plastic scintillators (1 mm thick × 15 mm ∅) were chosen as optimal-sized samples and designed to attach to a flat-faced phantom for irradiation using electron beams. Scintillator dosimeter response to radiation damage, dose rate, and temperature were studied. The effect of varying scintillator diameter and thickness on light output was evaluated. Furthermore, the scintillator emission spectra and impact of dosimeter thickness on surface dose were also quantified. Since the scintillators were custom-machined, dosimeter-to-dosimeter variation was tested. Scintillator surface dose measurements were compared to those obtained by optically stimulated luminescence dosimeters (OSLD).

Light output from scintillator dosimeters evaluated in this study was insensitive to radiation damage, temperature, and dose rate. Maximum wavelength of emission was found to be 422 nm. Dose reported by scintillators was linearly related to that from OSLDs. Build-up from placement of scintillators and OSLDs had a similar effect on surface dose (4.9% increase). Variation among scintillator dosimeters was found to be 0.3 ± 0.2%. Scintillator light output increased linearly with dosimeter thickness (~1.9 × /mm). All dosimeter diameters tested were able to accurately measure surface dose.

Scintillator dosimeters can potentially improve surface dosimetry-associated workflow for TSET in the radiation oncology clinic. Since scintillator data output can be automatically recorded to a patient medical record, the chances of human error in reading out and recording surface dose are minimized.

## Introduction

Surface dosimetry is widely used in radiotherapy. Specifically, in the case of total skin electron therapy (TSET), treatment efficacy is dependent on administration of a uniform radiation field across the body (Hensley *et al* 2013). Ensuring effective treatment delivery requires measurement and verification of surface dose (Kumar *et al* 1977, Desai *et al* 1988). Multiple systems which provide estimates of the delivered dose within a small surface area for verification of beam delivery are available commercially. The most common limitation of these devices is the need to keep track of where each probe is placed, since dose is read out and inserted into the patient record manually. The labor and time involved in this process comes at a high resource cost, and so while many centers have them, most limit their use because of the high level of human involvement in the processes (Butson *et al* 1996, Marre and Marinello 2004, Bufacchi *et al* 2007, Ben *et al* 2013, Desrosiers 2014, Schüttrumpf *et al* 2018).

There exists a clinical need for a completely electronic system which does not require manual manipulation, post-exposure processing, or coupling of dose and corresponding anatomical location into the patient record. Thus, this study examines a number of technical issues associated with measuring surface dose by imaging scintillation emission from plastic targets directly attached to human tissue and tissue-mimicking phantoms during TSET (Bruza *et al* 2018, Tendler *et al* 2018).

The main tools used for surface dosimetry have been thermoluminscent dosimeters (TLDs) or newer systems like optically stimulated luminescence dosimeters (OSLDs) (Yusof *et al* 2015, Bourland 2016). Even more recently, some centers have shifted to MOSFET, diode detectors or fiber coupled point scintillator detectors (Sengupta *et al* 2016, Fabregat Borrás *et al* 2017, Wang *et al* 2018). It is notable though, that the clinical value of surface radiation detectors remains high and continues to grow (Kry 2016, Beaulieu and Beddar 2016, Eyadeh *et al* 2017). Historically, TLDs (±5% error) (Kirby *et al* 1992, Allisy-Roberts and Williams 2008) and OSLDs (±3% error) (Reft 2009) have served as standards in the clinic even though their use can be affected by problems like high variation in dose estimates, manual location tracking error, and a laborious readout process. The value of diodes and scintillator fibers is that they are easier to place, and the readout is immediate after treatment. Recent development of fiber coupled scintillators offers smaller temperature, humidity and energy dependence than the previously mentioned methods (Beaulieu *et al* 2013, Beaulieu and Beddar 2016, Carrasco *et al* 2016, Dimitriadis *et al* 2017). However, like diodes, semiconductor and optical fiber-coupled dosimeters are placed on the patient skin with long cables, and the output values must still be entered into the patient record manually. Radiotherapy as a field, has been converging towards automated processes where tools should feed data to and from electronic systems seamlessly. Ideally, this could be accomplished with imaging systems where patient position and dosimetry information could be captured simultaneously—potentially enabling automation of the dose verification process. The scintillator whole-body imaging approach discussed in this study, combined with automated read-out, could meet this goal (Bruza *et al* 2018, Tendler *et al* 2018).

This scintillator-based surface dosimetry method was clinically deployed for monitoring surface dose in patients being treated with TSET (Bruza *et al* 2018, Tendler *et al* 2018). The technique is based on the linac-synchronized (time-gated) imaging of small plastic scintillating discs that are attached directly to the skin surface. Images are collected remotely, in real-time, and could be automatically saved to the patient medical record. Use of this novel dosimetry technique can potentially allow for time savings and improvement in clinical dosimetry-related workflow. Importantly, it was shown that the scintillation images can accurately report surface dose for TSET delivery, when compared against standard OSLD dosimeters. As such, this data has served as motivation for this study (Tendler *et al* 2018).

The primary purpose of this current study was to evaluate the physical characteristics of scintillator dosimeters that are essential for clinical TSET utilization. Namely, we characterize the emission spectrum, dependence on temperature, dose rate, radiation damage, and the influence of thickness and diameter on light output. Given that each dosimeter is custom-machined, scintillator-to-scintillator variation was also evaluated. Considering that these plastic dosimeters are placed directly on the skin surface in the path of the beam, the impact of dosimeter thickness on deposited surface dose was quantified.

## Methods

### Scintillator dosimeters and imaging setup

Scintillators were custom-manufactured by Elijen Technogies. The disc-shaped dosimeters are composed of EJ-212 plastic and painted with EJ-510 reflective paint (Elijen Technologies, Sweetwater, TX) on both the rear face and edge. The TSET scintillators measured 1 mm in thickness and 15 mm in diameter, with these dimensions being chosen based upon previous tests of the ability to resolve them with the camera system. Scintillation emission was captured by an intensified CMOS camera, C-Dose (DoseOptics LLC, Lebanon NH), whose intensifier was gated to signal from the linac klystron (KLYI, 4.5 *μ*s intensifier gate pulse width). A gate delay of 1.7 *μ*s and 100 *μ*s was used for scintillation and background image acquisition, respectively. The images were captured at a frame rate of 11 fps. Considering a standard 360 Hz linac pulse repetition rate and alternating scintillation/background frame acquisition, each frame was composed of accumulation of 16 scintillation pulses. The camera was placed behind and to the side of the gantry head at a camera-to-scintillator distance of 4 m. A fiber optic communication cable was routed to a computer located outside of the treatment vault. Images are collected using online background and darkfield subtraction; online median and spatial filters are also utilized. These image processing steps have been previously described in detail (Bruza *et al* 2018, Tendler *et al* 2018).

**Figure 1.**
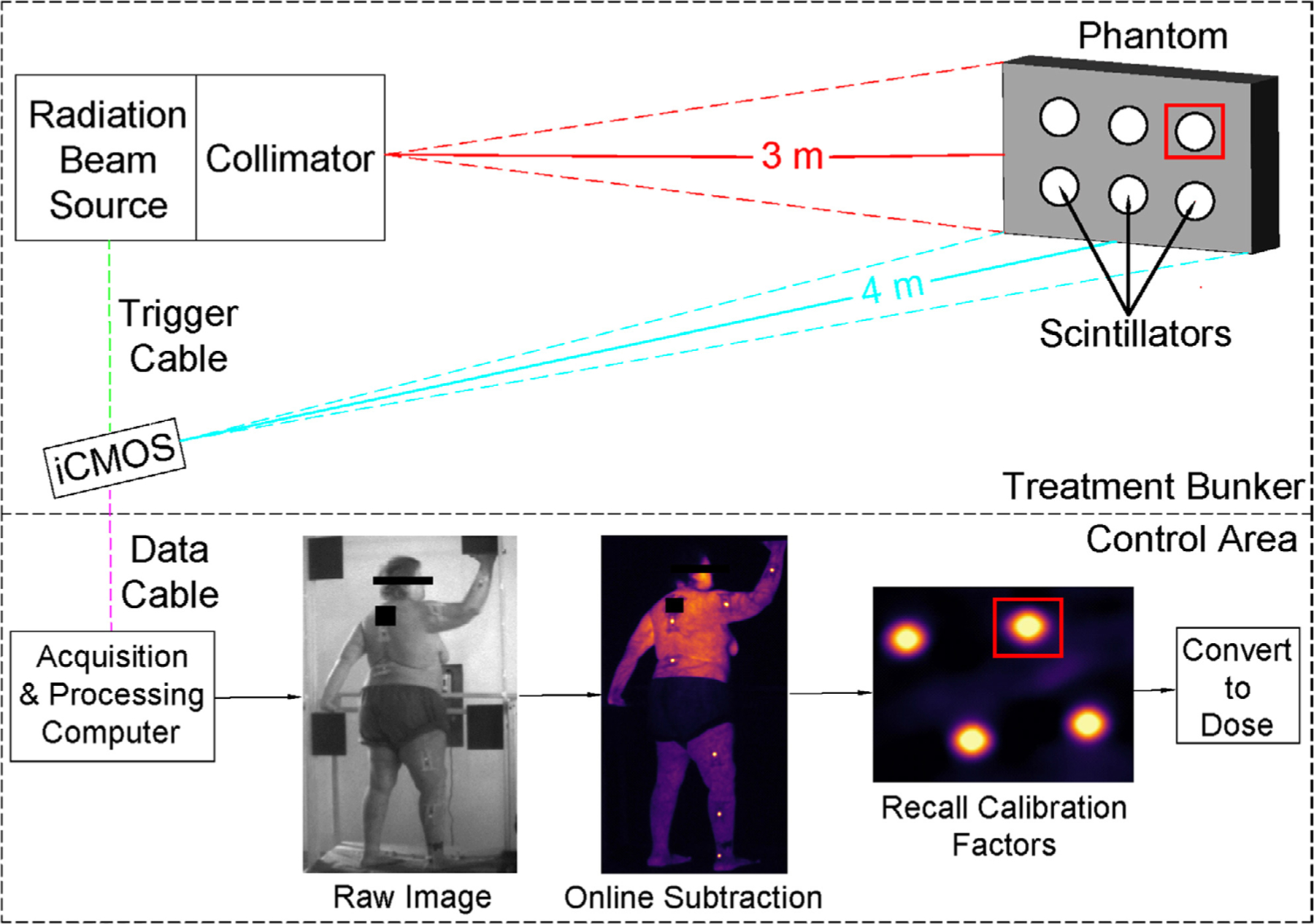
An illustration of the TSET imaging setup. The linac is represented by the ‘radiation beam source’ and corresponding ‘collimator’. Image acquisition is time-gated to linac pulses; thus, a triggering cable is attached between the signaling panel of the linac and the camera (‘trigger cable’). Image data is sent to a computer located outside of the treatment bunker via optical data cable. Overview of internal image processing steps is shown in ‘control area’ panel. Sample ROI are shown as red squares.

A Varian 2100 CD linac (Varian Medical Systems, Palo Alto, CA) was used for irradiation during all characterization studies. The experimental setup used to collect phantom measurements in this study was identical to that previously utilized for patient imaging (Tendler *et al* 2018). TSET treatments were delivered with a High Dose Total Skin Electron protocol (HDTSe, 6 MeV @ 66 cGy min^−1^, measured at the patient). This same procedure was used to irradiate samples at a source-to-surface distance (SSD) of 3 m for several of the studies, referred to as the TSET setup. Figure 1 presents a schematic of the entire imaging setup.

### Dose estimation from scintillator intensity values

The dose estimation process fits an analytical model of scintillator emission to experimental data within a region of interest (ROI) using a minimization algorithm. This technique assumes that scintillator radiance is proportional to imparted dose (Birks 1964). The model considers a case where photon emission of an arbitrarily angled scintillator results in a scaled, rotated, ellipsoidal disk. Importantly, the pixel intensity amplitude *b* of this disk is proportional to scintillator radiance.


(1) $$F=\left\{\begin{array}{ll} b & \text{ if }\frac{{\left(\left[x-x_{\text{c}}\right]\;\text{cos}\;\theta +\left[y-y_{\text{c}}\right]\;\text{sin}\;\theta \right)}^{2}}{w^{2}}+\frac{{\left(\left[x-x_{\text{c}}\right]\;\text{sin}\;\theta +\left[y-y_{\text{c}}\right]\;\text{cos}\;\theta \right)}^{2}}{h^{2}}⩽1\\ 0 & \text{otherwise} \end{array}\right\}.$$

The scintillator model function *F* can then be expressed as:

where *x*(*m*) and *y*(*n*) are pixel coordinates (indices (*m*,*n*) of the pixels in the image ROI), (*x*_c_, *y*_c_) are centroid coordinates; *w*, *h*, and *θ* are width, height, and angle of the ellipse. In addition, to account for image blur-ring due to camera and lens imperfections, a 2D Gaussian point spread function *G* is considered with standard deviation *σ*:

(2) $$G=\frac{1}{2\pi \sigma^{2}}\text{exp}\left(-\frac{x^{2}}{2\sigma^{2}}-\frac{y^{2}}{2\sigma^{2}}\right).$$

Thus, the final model function takes the following form:

(3) M=a+(b∗G)

where *a* is an offset value due to Cherenkov emission from the skin, *b* is the amplitude of the fitted scintillator model function, and * denotes a multiplication operation. As mentioned above, amplitude *b* is a scaled value of scintillator radiance and is expected to be directly proportional to the dose *d*, received by the scintillator:

(4) $$d=k_{\text{c}}b.$$

The factor *k*_c_ is derived from a calibration scintillator image in which reference dose measurements are acquired concurrently using an OSLD, as described in section 2.1 below. The fitting algorithm, implemented in MATLAB (Mathworks, Natick, MA), finds the best fit of the model function (3) to the observed image intensities (pixel-wise) *I*_*m*,*n*_ by minimizing the error functional *χ*^2^, representing the sum of the squared residuals:

(5) $$\chi^{2}=\underset{m,n}{\sum }{\left(I_{m,n}-M\left(b,\beta_{\text{psf}}\right)\right)}^{2}.$$

*β*_psf_ = [*x*_c_, *y*_c_, *w*, *h*, *a*, *b*, *σ*, *θ*] is a vector that holds the fitting parameters where psf represents ‘per scintillator and image frame’.

The dose estimation workflow goes as follows:

1. The patient image stack is imported and flat-field corrected.
2. Approximate coordinates of the centroid of each scintillator are entered.
3. Initial guesses and bounds for *β* corresponding to the first frame of the image stack are entered.
4. The model function *M* is fit to pixel intensities within a pre-defined scintillator ROI—established using (*x*_c_, *y*_c_) coordinates—per image frame. The optimization algorithm utilized is trust-region reflective with a convergence tolerance of 1 × 10^−10^.
5. The resulting *β* produced per frame are extracted.
6. To improve the accuracy of fitting algorithm, step 4 is repeated. However, now the mean values of each fitting parameter (obtained from the matrix of *β*s in step 5) are used as the initial guess and bounds.
7. Upon completion, final values of *b* are summed across all frames, per scintillator, and corrected by *k*_c_ yielding the cumulative dose per scintillator—herein referred to as scintillator output.

The parameters of *β* are computed on a per frame basis. This greatly reduces the impact of motion artifacts, which are inevitable and occasionally rather large (~10 cm s^−1^ observed) in a typical TSET session. The size of the scintillator ROI (user-selected and fixed) in our measurements was 40 × 40 pixels, corresponding to a physical dimensions of 1.5 × 1.5 cm. Sample outlines of the ROI are shown as red boxes in figure 1, an ROI is created per scintillator and frame; thus, the number of ROIs in each frame will correspond to the number of scintillators. Therefore, a dosimeter can move approximately 1 cm between frames without necessitating reassignment of the centroid ROI. The motivation for selecting these functions (equations (1)–(5)) was based on minimizing the effects of angular dependency between incident radiation, scintillator discs, and the camera. By utilizing the function described in equation (1), surface dose can be computed with no angular dependency in the angles of 0°–55° (Tendler *et al* 2018). In earlier versions of this research, scintillator light emission was related to surface dose by: (1) Thresholding the cumulative scintillator image (2) selecting and ROI inside the scintillator (3) taking the average of the pixel intensities in the ROI. A number of correction factors (lens throughput, camera-scintillator angle, etc) were then applied to yield the energy fluence within the scintillator ROI (Bruza *et al* 2018). See supplemental material (stacks.iop.org/PMB/64/125025/mmedia) for MATLAB image analysis code.

## 1. Analysis of scintillator physical properties

### 1.1. Impact of temperature on scintillator output

**Figure 2.**
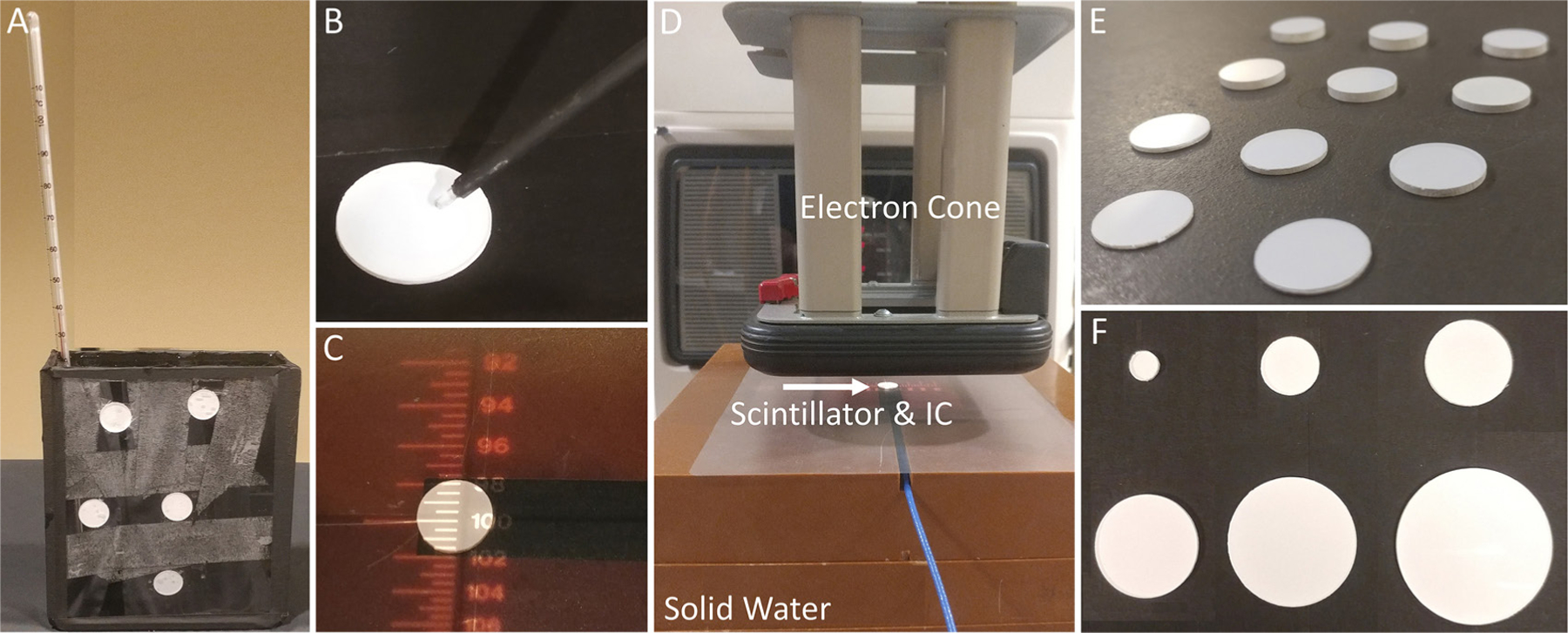
(A) Setup for temperature verus scintillator light output (B) spectrometer fiber optic probe setup (C) scintillator placed on-top of active volume of IC (D) set-up for testing effect of scintillator thickness on surface dose. Solid water, electron cone, and scintillator placed on-top of active volume of IC are labeled (E) scintillators of varying (0.65–3.13 mm) thickness (F) scintillators of varying (5–30 mm) diameters.

Scintillators (*n* = 4, thickness = 1.08, 1.09, 1.09, and 1.09 mm, diameters = 1.5 cm) were mounted on the custom phantom shown in figure 2(A) and 600 ml of water was added. The phantom was placed at a 3 m SSD and 4 m away from the camera. The temperature of the water was measured using a Fisherbrand 150415C thermometer (Fisher Scientific, Hampton, NH). Initially, 10 °C water was added to the container and stirred to ensure temperature homogeneity throughout the phantom. Data was obtained by using the TSET imaging setup and administering 300 monitor units (MU). Temperature of the water was increased by placing the phantom on a hot plate (kept at a constant 45 °C), again, the water was stirred during this process. Scintillator output data was obtained at temperatures of 15, 20, 25, 30, 35, 37, and 40 °C.

### 1.2. Scintillator emission spectra

The emission light spectra of the scintillators was recorded using an Ocean Optics USB4000 Spectrometer (Halma PLC, Amersham, UK). A scintillator was attached to a flat-faced phantom and irradiated with 6 MeV using the TSET imaging setup with 100 MU. The spectrometer probe was placed such that the tip was nearly touching the surface of the scintillator (figure 2(B)). A 5 s exposure time was used for acquisition. Background signal was collected by acquiring data with no active radiation field. Furthermore, native signal (Cherenkov and scintillation) generated in the fiber optic probe itself was evaluated by removing the scintillator and taking a measurement while irradiating the fiber optic only. Native spectra were obtained for a 6 MeV beam. These spectra were then subtracted from the scintillator emission data and the results were smoothed using a Savitzky–Golay filter in MATLAB.

### 1.3. Scintillator light output versus dose rate

To determine whether dose rate of the incident beam influences scintillator output, *n* = 5 scintillators were attached to a flat-faced phantom and imaged using the TSET setup. Dose rates of 100, 200, 300, 400, 500, 600, and 1000 MU min^−1^ were used to irradiate samples to 300 MU. Scintillator output was normalized to the mean of all values obtained in this experiment.

## 2. Scintillator performance

### 2.1. Scintillator-to-scintillator variation and calibration factor

Dosimeter variation was evaluated by comparing scintillator-to-scintillator output from a batch of 29 TSET scintillators. Given that each scintillator was custom machined and painted, thickness and diameters of scintillators ranged from 1.04 to 1.09 mm and 14.9 to 15.1 mm, respectively. The scintillators were mounted to a flat-faced phantom and imaged using the TSET geometry and exposed to a dose of 500 MU. Scintillator output was computed per dosimeter; percent difference from the mean output value was calculated for each scintillator. In addition, following previously described methodology, this experiment was re-run to obtain a TSET scintillator calibration factor for converting dosimeter light output to dose (Tendler *et al* 2018). Six OSLDs were attached to the phantom directly adjacent to scintillators and exposed to doses of 25–200 cGy. A linear trend line was fit to a plot of average dose reported by OSLD verus scintillator output.

### 2.2. Scintillator output versus lifetime radiation exposure

To determine whether scintillator light output is influenced by lifetime exposure to radiation, 4 new (previously unexposed to radiation) TSET scintillator dosimeters were subjected to doses of 0, 1300, 2700, 4100, 8500, and 15 000 Gy. Scintillators were first irradiated with 300 MU using the TSET imaging setup to obtain a baseline output reading. Then, scintillators were placed at 60 SSD and irradiated to the doses listed above. After each intended dose was administered, the dosimeters were placed in the TSET imaging setup and irradiated with 300 MU to obtain incremental scintillator output measurement. Following completion of this test, scintillator output was re-verified after a period of 20 d (dosimeters were kept in a radiation-free environment) by using the TSET imaging setup and irradiating with 300 MU.

### 2.3. Impact of scintillator thickness on surface dose

Scintillators of different thicknesses (0.65, 0.80, 1.08, 1.34, 1.51, 1.75, 2.11, 2.28, 2.57, 2.85, and 3.13 mm) were placed directly on top of a calibrated PTW 23342 ionization chamber (IC) (PTW, Freiburg, Germany). Scintillator samples of varying thickness are shown side-by-side in figure 2(E). It should be noted that this specific chamber has a sensitive volume with a diameter of 3 mm which is smaller than the 15 mm diameter of the TSET scintillators—the disc was able to completely cover the entrance window of the IC, figure 2(C). A stack of 10 cm of solid water was used to provide backscattering, the top-most block had a cut-out insert for the IC—the surface of the chamber was flush with the top of the stack figure 2(D). Scintillators were irradiated at a SSD of 100 cm with a 10 cm × 10 cm field using a 6 MeV for 100 MU (1000 MU min^−1^). For reference, the effect of an optically stimulated luminescence detector (nanoDOT, Landuer Inc. Glenwood, IL) on surface dose was also evaluated. The percent change in surface dose caused by placing an OSLD and scintillators with varying thicknesses on-top of an IC were computed.

### 2.4. Scintillator output versus thickness

To evaluate the impact of dosimeter thickness on scintillator output, scintillators used in section 2.3 were mounted to a flat-faced phantom and irradiated with 300 MU using the TSET imaging setup. Scintillator output was obtained for each dosimeter. Data was normalized to output from the thinnest (0.65 mm) dosimeter to determine how relative output changes with thickness.

### 2.5. Scintillator output versus diameter variation

Scintillators of 1 mm thickness and diameters of 5, 10 15, 20, 25, and 30 mm were attached to a flat faced phantom, figure 2(F). OSLDs were also attached to the phantom directly adjacent to scintillators to provide reference surface dose measurements. The phantom was irradiated using the TSET imaging setup with doses of 100, 300, 500, 700, 900, and 1100 (MU) corresponding to 31, 67, 104, 144, 182, and 223 cGy, as measured by OSLD. Scintillator output was compared to surface dose measured by OSLD.

## 3. Results

### 3.1. Scintillator physical properties

#### 3.1.1. Impact of temperature on scintillator output

**Figure 3.**
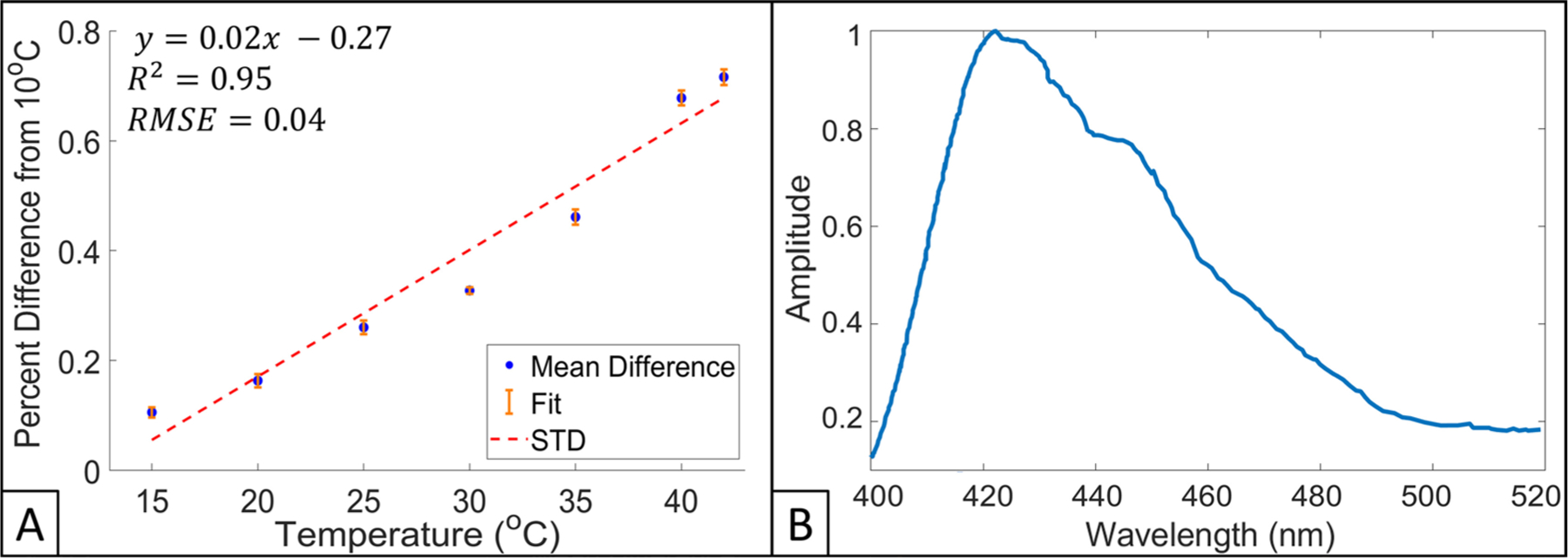
(A) Scintillator output as a function of temperature, values are presented as a percent difference from a baseline measurement obtained at 10 °C for 6 MeV electrons. Equation for linear fit (red dotted line), *R*^2^, standard deviation (STD, orange colored) bars, and RMSE are also presented. (B) Scintillator emission spectra for 6 MeV electron beam.

Scintillator output was evaluated over a range of temperatures (10, 15, 20, 25, 30, 35, and 40°) for *n* = 5 TSET scintillators using 6 MeV. For a change in temperature of 30 °C (10 °C–40 °C) it was found that average output increased by 0.69 ± 0.02%. Scintillator output near body temperature (35 °C), compared to room temperature (~25 °C), was found to be 0.21 ± 0.03% higher on average. Figure 3(A) presents data for average scintillator output (normalized percent difference to the baseline output at 10 °C) across the tested temperature range. A linear fit was generated for this data set with an *R*^2^ = 0.95 and RMSE = 0.05, shown in figure 3(A).

#### 3.1.2. Scintillator emission spectra

The emission light spectra of the TSET scintillators when irradiated with 6 MeV electrons is shown in figure 3(B). The scintillators were found to have a maximum wavelength of emission of 422 nm; emission spectra were normalized to this wavelength. Scintillator spectra had ⩾20% light emission in the bandwidth of 403–492 nm.

#### 3.1.3. Scintillator light output versus dose rate

**Figure 4.**
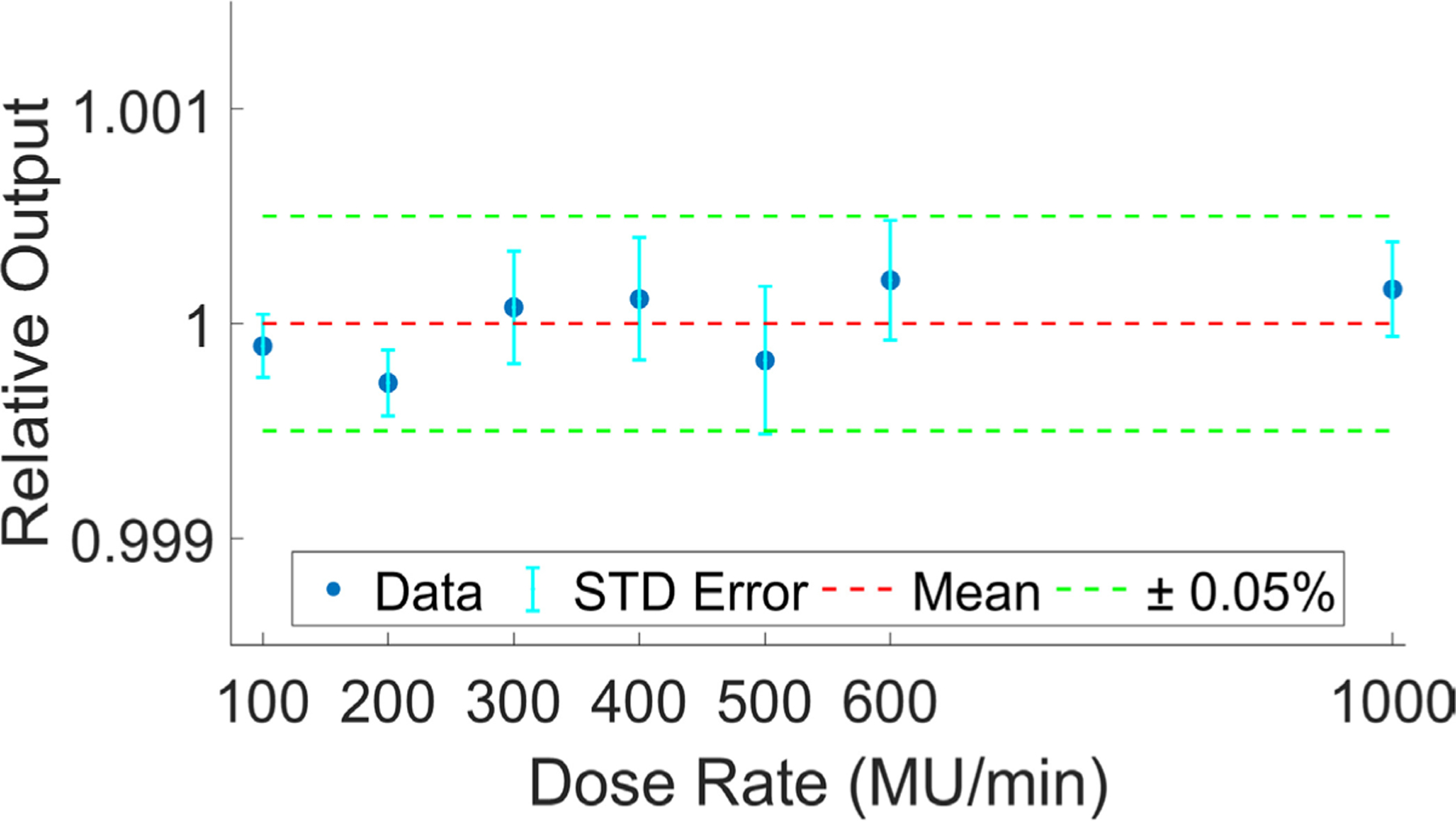
Relative light output versus dose rate. Each data point (blue) shows an average of *n* = 5 scintillators with corresponding standard deviation error bars (cyan). Mean and ±0.05% for all data points is shown in red and green, respectively.

The relationship between dose rate and cumulative scintillator light output is shown in figure 4. Scintillator output was normalized to the mean of all measured data points in this experiment, depicted as a horizontal red dotted line. Each data point represents an average of the *n* = 5 scintillators utilized in this experiment, 1 standard deviation error bars (vertical cyan colored) are also provided in this figure. Scintillators reported data within 0.05% of the mean across all dose rates tested—dose rate had no impact on scintillator light output.

### 3.2. Scintillator performance

#### 3.2.1. Scintillator-to-scintillator variation and calibration factor

**Figure 5.**
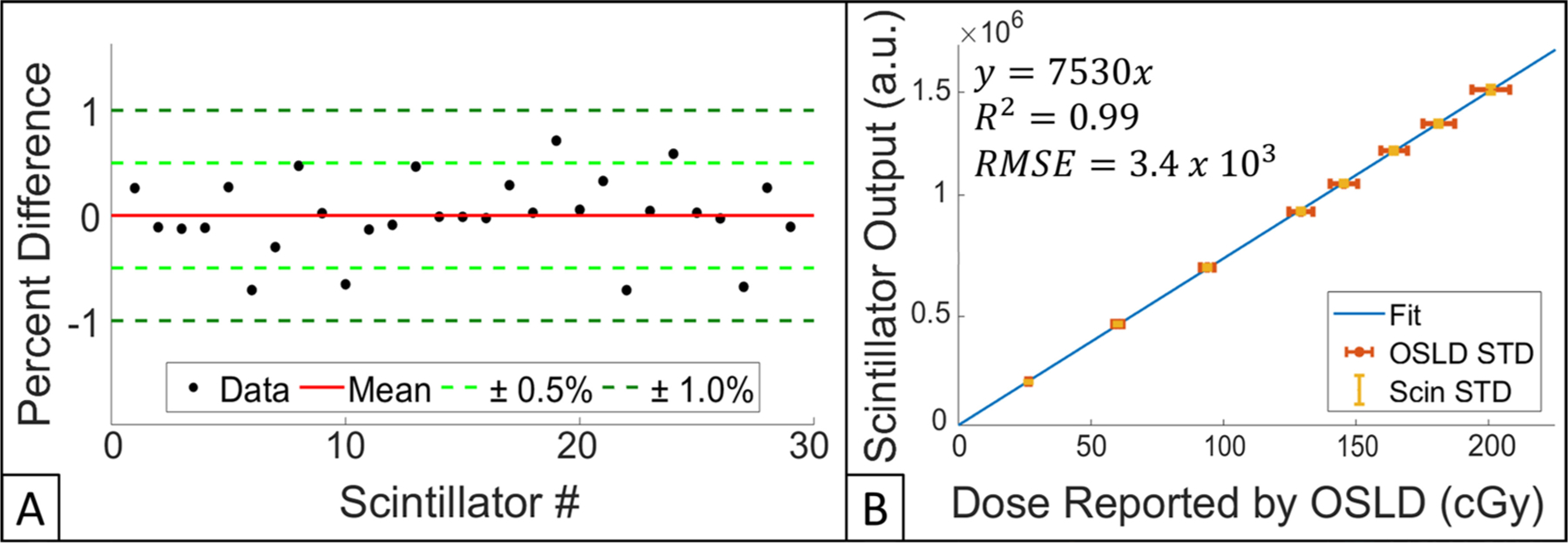
(A) Scintillator-to-scintillator variation for 29 dosimeters. Mean of all percent differences, ± 0.5% and ± 1.0% are shown red, light green, and dark green, respectively. (B) Scintillator output verus dose reported by OSLD. Linear fit, OSLD STD, and scintillator STD are shown in blue, red and yellow, respectively. Equation for trendline, *R*^2^, and RMSE are also provided.

For a group of *n* = 29 TSET scintillator dosimeters, output per scintillator is shown as percent difference from mean, per scintillator, in figure 5(A). It was found that the mean percent difference was 0.3% ± 0.2%; 29/29 and 23/29 scintillators had output within 1% and 0.5% of the mean, respectively.

Upon plotting scintillator output for all 29 dosimeters versus dose reported by OSLD (range of exposures were 20–200 cGy), a linear fit was calculated (*R*^2^ = 0.99) and a dose conversion calibration factor of 7530 a.u. cGy^−1^ was derived, figure 5(B).

#### 3.2.2. Scintillator output verus lifetime radiation exposure

**Figure 6.**
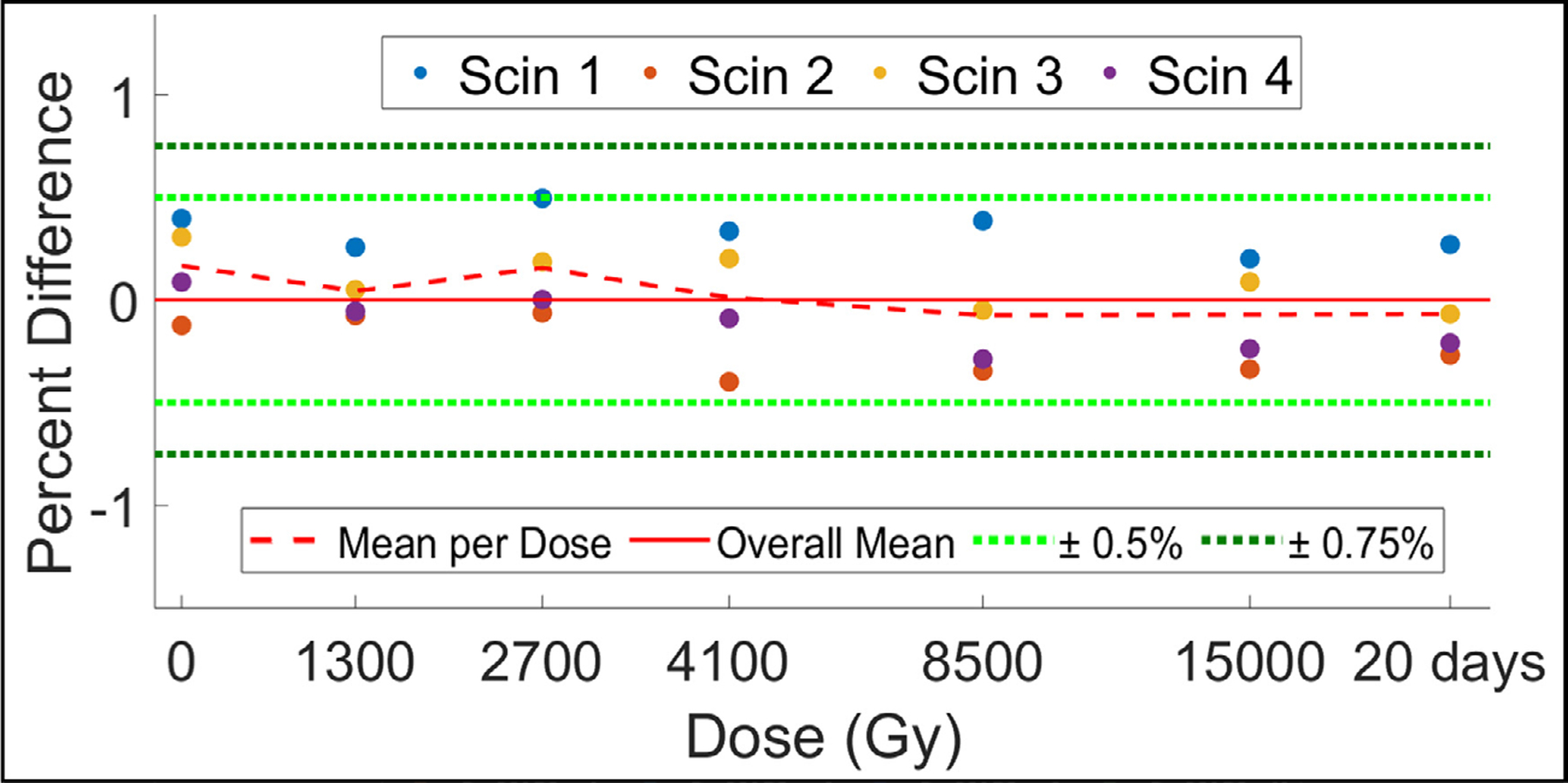
Scintillator output for a group of 4 new scintillators after being exposed to a cumulative dose of 15 000 Gy. Data is presented as percent difference from overall mean of all data collected (solid red line). A verification measurement taken 20 d post-15 000 Gy exposure is also shown. Mean percent difference from overall mean per exposed dose is displayed as a dotted red line. ±0.5% and ±1.0% are in dotted light and dark green lines, respectively.

To determine whether cumulative radiation exposure influences scintillator light output, *n* = 4 TSET scintillators were exposed to a total of 15 000 Gy. Figure 6 shows scintillator output as a function of total radiation exposure. Compared to scintillators having received no prior dose, scintillators showed an average 0.2% decrease in signal output after exposure to 15 000 Gy. Furthermore, the same batch of dosimeters underwent verification testing 20 d post-15 000 Gy exposure, less than an average 0.1% difference in scintillator output was noted. Given that we have previously shown that the camera system was stable within 2% ± 1% over a 6-month testing period (Tendler *et al* 2018), we can reasonably say that there was no detectable impact of radiation exposure on scintillator output.

#### 3.2.3. Scintillator thickness verus surface dose

**Figure 7.**
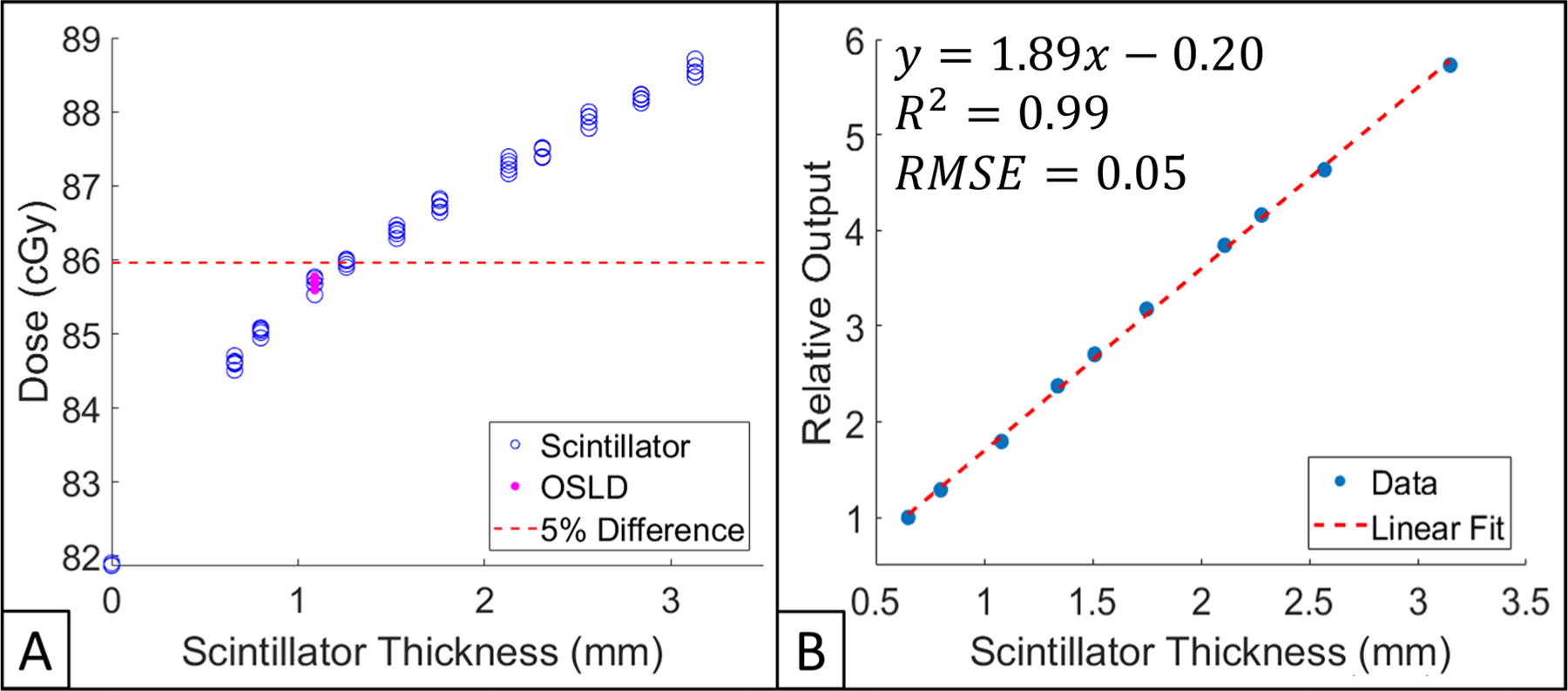
(A) Impact of scintillator thickness on surface dose measured by IC. All data points are normalized to IC reading obtained with no dosimeter present (baseline). 5% from baseline is shown as a dotted red line. IC measurements obtained with scintillators and an OSLD place upon the IC are shown in hollow blue circles and filled pink dots, respectively. (B) Scintillator light output as a function of thickness. All data points are normalized to the thinnest dosimeter (0.66 mm). Linear fit, as well as corresponding *R*^2^ and RMSE are shown.

Scintillators of different thicknesses were placed directly on-top of an IC to evaluate the effect of increasing scintillator thickness on surface dose (figure 7(A)). For scintillators of thicknesses ranging from 0.66 mm (thinnest tested) to 3.13 mm (thickest tested), surface dose was found to increase by an average of 2.6% and 6.6%, respectively, when compared to a baseline reading with no scintillator present. The impact of TSET scintillators (1 mm thick) on surface dose was compared to that of a standard nanoDot OSLD (2 mm thick), both dosimeters increased surface dose, on average, by 4.9%. All scintillator thickness <1.26 mm increased surface dose by <5%.

#### 3.2.4. Scintillator output verus thickness

The relationship between scintillator thickness and normalized output is shown in figure 7(B). By normalizing dosimeter output to the thinnest scintillator (0.66 mm), it was found that scintillator output follows a linear trend with an *R*^2^ = 0.99. Thus, for every mm increase in scintillator thickness, output increase by approximately 1.9 times—compared to the thinnest scintillator, the thickest scintillator (3.13 mm) had 5.7 times greater output.

#### 3.2.5. Scintillator output verus diameter

**Figure 8.**
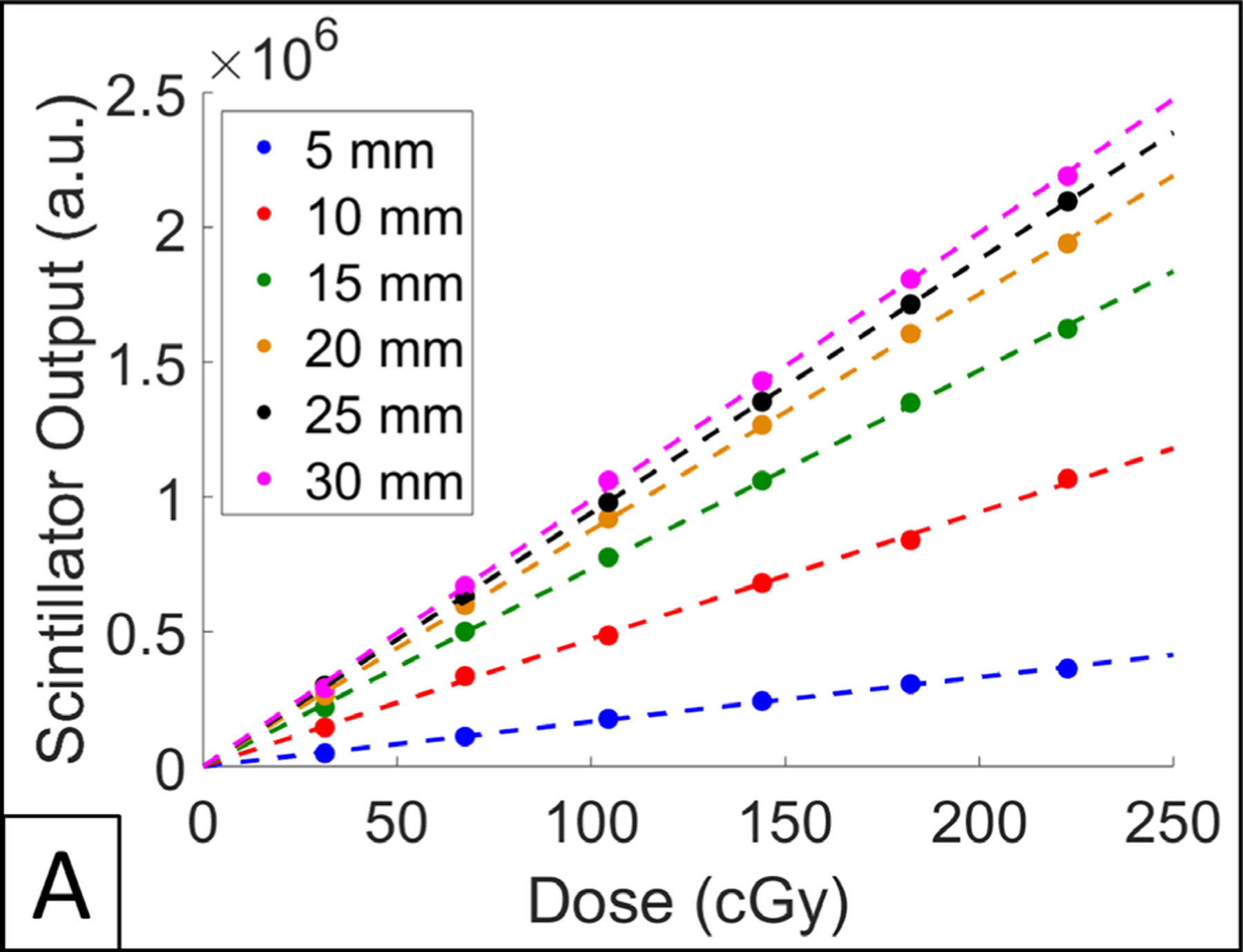
(A) Scintillator output over a range of doses for varying dosimeter diameters.

Scintillator output, as a function of exposed dose, for dosimeters of varying diameters (thickness of 1 mm) is shown in figure 8. The custom fitting algorithm was able to successfully produce values for scintillator output in all dosimeters tested; all dosimeters responded to increased amounts of dose in a linear fashion. It should be noted that scintillator output was converted to dose by using surface dose measurements reported by OSLDs. The slopes of the curves reported in figure 8 correspond to calibration factors for dosimeters of varying diameter.

## 4. Discussion

The purpose of scintillator dosimeters is to provide a surrogate dose measurement that enable estimation of absolute dose at a point of interest. We have shown that the response of scintillator dosimeters is independent of dose rate, radiation damage, and temperature, within clinically relevant range of values. Furthermore, TSET scintillators were found to have a similar impact on surface dose compared to OSLDs, a standardized surface dosimetry technique. We have previously demonstrated that within the context of TSET, scintillator dosimeters are distance and angle independent (Tendler *et al* 2018). The current manufacturing methodology has been able to produce dosimeters with sufficiently low (0.3% ± 0.2%) scintillator-to-scintillator variation; thus, a bulk calibration factor can be utilized for light output to dose conversion.

The emission spectra and light output response to changes in thickness and diameter of scintillators is presented above. Thus, knowing this information, one could adapt the scintillator dosimetry system for situations where a smaller treatment field (with large dose gradients) is utilized. For example, dosimeter diameter and thickness can be reduced, the camera system, meanwhile, can be modified to have higher sensitivity to scintillator light output.

The addition of automatic scintillation detection capability to the acquisition software provided with the C-Dose camera system is currently under development. This technique will utilize computer vision algorithms to assess properties of potential scintillator locations (based on a range of thresholded images), including intensity, area, circularity, inertia, and convexity. Scintillation centroid locations are thereby automatically determined in each frame and tracked over time through the course of treatment. The fitting model is then used to determine dose values in cGy from scintillation intensity accumulation. In the case of false positive or negative identification of scintillators, the user can add or remove locations, respectively.

To eliminate the need for individually wrapping scintillators in cellophane, testing is underway to create a protective coating for scintillator dosimeters. Combined with their resistance to radiation damage (tested up to 20 000 Gy), we envision these dosimeters have the potential for long-term reusability. The protective coating would enable dosimeter cleaning with alcohol-based chemical agents in between uses and prevent scratching or chipping of the reflective paint.

In addition to TSET, these dosimeters can be used in estimating surface dose during treatment scenarios where static open fields are utilized. For example, there is utility in estimating surface dose during clinical situations such as electron fields. Due to their modest size and stable dose response, scintillator dosimeters can be used to practically and efficiently provide this information.

## 5. Conclusion

This report serves as reference material for the time-gated imaging of scintillator dosimeters in determining surface dose of patients undergoing TSET. Combined with a previous successful human pilot trial (Tendler *et al* 2018), the data presented in this study shows that scintillator dosimeters are a viable alternative to currently existing surface dosimetry techniques.

## Supplementary Material

Supplementary Information 2

Supplementary Information 1 1
