# Supplementary material for: Characterization of a non-contact imaging scintillator-based dosimetry system for total skin electron therapy
Source: Phys Med Biol. Author manuscript; Available in PMC 2023 Nov 16. (PMC10653344; doi:10.1088/1361-6560/ab1d8a)
Supplement: Supplementary Information 2 [file NIHMS1944085-supplement-Supplementary_Information_2.docx]

%% Characterization of a Novel Scintillator-Based Surface Dosimetry System for Total Skin Electron Therapy

% Code for ellipse-convolved Gaussian function

% Irwin Tendler, Petr Bruza, Mike Jermyn, Xu Cao, Benjamin Williams, Lesley Jarvis, Brian Pogue, David Gladstone

%% begin function

function g = ellipseFcn(A,X)

% Set bounds

% amplitude = peak of gaussian

% A = [xc,yc,a,b,amplitude,offset,thetasigma]

x = X(1,:,1); % x-values of mesh grid

y = X(:,1,2); % y-values of mesh grid

xc = A(1); % xc = x-coordinate of centroid

yc = A(2); % yc = y-coordinate of centroid

a = A(3); % a = width of ellipse

b = A(4); % b = width of ellipse

amplitude = A(5); % amplitude = peak of gaussian

offset = A(6);

theta = A(7); % theta = ellipse angle (radians)

sigma = A(8); % signma = gaussian blur width

g = zeros(size(X,2),size(X,1));

% apply function

for m = 1:size(g,1)

for n = 1:size(g,2)

if ( (((x(m)-xc)*cos(theta) + (y(n)-yc)*sin(theta))^2)/a^2 + (((x(m)-xc)*sin(theta) - (y(n)-yc)*cos(theta))^2)/b^2 ) <= 1

g(n,m) = 1;

end

end

end

g = imgaussfilt(g,sigma,'Padding','replicate','FilterDomain','spatial');

% scale output

g = amplitude .* g + offset;
