# Supplementary material for: Characterization of a non-contact imaging scintillator-based dosimetry system for total skin electron therapy
Source: Phys Med Biol. Author manuscript; Available in PMC 2023 Nov 16. (PMC10653344; doi:10.1088/1361-6560/ab1d8a)
Supplement: Supplementary Information 1 1 [file NIHMS1944085-supplement-Supplementary_Information_1_1.docx]

%% Characterization of a Novel Scintillator-Based Surface Dosimetry System for Total Skin Electron Therapy

% Code for converting scintillator output to dose

% Irwin Tendler, Petr Bruza, Mike Jermyn, Xu Cao, Benjamin Williams, Lesley Jarvis, Brian Pogue, David Gladstone

%% Clear Matlab

clear all

close all

clear folders

%% Part 1: Import data

label = 'XXX'; %create label for data in Part 3

% import data

% depends on naming convention

ch_filename = char(strcat(folders(1,:),'XXXX',ch_out_str(1),'.TIFF'));

ch_info = imfinfo(ch_filename);

ch_tiff = Tiff(ch_filename,'r');

[frames,~] = size(ch_info);

for n=1:frames

ch_tiff.setDirectory(n);

ch(:,:,n) = double(ch_tiff.read()); %ch contains data

end

[Height,Width, CH_frames] = size(ch); % obtain size info

%% Part 2: Apply flatfield (FF) correction

ff_name = 'normal_FF.tif'; % input name of normalized FF

% import FF file

ff_tiff = Tiff(ff_name,'r');

ff = double(ff_tiff.read());

ff = repmat(ff, [1,1,size(ch,3)]);

ch = ch ./ ff; % apply flatfield

%% Part 3: Create data table

% Assume in this example there are only 2 scintillator locations

position = {strcat('upper_',label),'Location 1','Location 2';'x-coordinate','','';

'y-coordinate','','';'Output','',''};

% x-coordinates of scintillator centroids

position{2,2} = XXX;

position{2,3} = XXX;

% y-coordinates of scintillator centroids

position{3,2} = XXX;

position{3,3} = XXX;

%% Part 4: Extract ROI and create numerical grid

cm = 20; % pixel width across 1/2 size of scintillator ROI

% set full ROI using previously defined centroids

S = ch(position{2,2}-cm:position{2,2}+cm,position{3,2}-cm:position{3,2}+cm,10);

% extract ROI

[n,m] = size(S);

% Numerical grids

[x,y]=meshgrid(-cm:cm,-cm:cm); X=zeros(m,n,2); U=zeros(m,n,2);

k(:,:,1)=x; k(:,:,2)=y;

%% Part 5: Set bounds and fitting parameters

% Set bounds

% xc = x-coordinate of centroid

% yc = y-coordinate of centroid

% a = width of ellipse

% b = width of ellipse

% amplitude = peak of gaussian

% offset

% theta = ellipse angle (radians)

% signma = gaussian blur width

% A = [xc,yc,a,b,amplitude,offset,thetasigma]

% [xc,yc,a,b,amplitude,offset,theta,sigma]

lb = [-10, -10, 2, 2, 1, 0, 0, 1]; % lower bound

ub = [10, 10, 10, 10, 5000, 2*median(S(:)), pi, 5]; %upper bound

% Inital parameters

A0 = [1, 1, 5, 5, 1000, median(S(:)), 1, 1];

%preallocation for results

A_stack = double(zeros(size(A0,2),CH_frames));

res_stack = double(zeros(m,n,CH_frames));

% Fit parameters

InterpMethod = 'linear'; % 'nearest','linear','spline','cubic'

FitOrientation = 'fit'; % 'fit': fit for orientation, 'dont' fit for orientation

options = optimoptions('lsqcurvefit','FiniteDifferenceStepSize', 0.5,...

'FiniteDifferenceType','forward','StepTolerance',1e-1,'FunctionTolerance',1e-10);

options.MaxFunctionEvaluations = 1000;

%% Part 6: Apply Fit

for j = 2:XX % XX depends on number of scintillators being analyzed

% approximate (x,y) coordinates of the scintillator centroid

coor = [position{2,j} position{3,j}];

for z = 1:size(ch,3) % applies fit per frame

S = ch(coor(2)-cm:coor(2)+cm,coor(1)-cm:coor(1)+cm,z); % extract ROI

%apply fit

[A,resnorm,res,flag,output] = lsqcurvefit(@ellipseFcn,A0,k,S,lb,ub,options);

% ellipseFcn is a custom function, ellipse-convolved Gaussian

% A matrix is described above

% resnorm = normalized residuales

% res = residual

% flag = exitflag (reason solver stopped)

% output = information about the optimization process

A_stack(:,z) = A; % save A-matrix per frame

end

A_stack_all(:,:,j) = A_stack; % save all A-matrices per scintillator

end

%% Part 7: Optimize Fit: Second Run-Through

%take mean values of A-matrices for all frames and scintillators

theta_ave = mean(A_stack_all,2);

% initialize fit in the same manner as before

% change initial guess to mean of A-matrix values generated in Part 6

for j = 2:XX

% approximate (x,y) coordinates of the scintillator centroid

coor = [position{2,j} position{3,j}];

% set ROI

S = ch(position{2,2}-cm:position{2,2}+cm,position{3,2}-cm:position{3,2}+cm,10);

% lower (lb) and upper (ub) bound guesses

lb = [-10, -10, theta_ave(3,1,j),theta_ave(4,1,j), 1, 0,...

theta_ave(7,1,j), theta_ave(8,1,j)];

ub = [10, 10, theta_ave(3,1,j),theta_ave(4,1,j), 5000, 2*median(S(:)),...

theta_ave(7,1,j), theta_ave(8,1,j)];

% Inital guess parameters

A0 = [1, 1, theta_ave(3,1,j),theta_ave(4,1,j), 1000,...

median(S(:)),theta_ave(7,1,j), theta_ave(8,1,j)];

for i = 1:size(ch,3) % applies fit per frame

S = ch(coor(2)-cm:coor(2)+cm,coor(1)-cm:coor(1)+cm,z); % extract ROI

%apply fit

[A,resnorm,res,flag,output] = lsqcurvefit(@ellipseFcn,A0,k,S,lb,ub,options);

A_stack(:,z) = A; % save A-matrix per frame

end

%sum max amplitude of fit of across frames for each scintillator

position{4,j} = sum(A_stack(5,:),2);

end

%%

% Acknowledgement: M. Diaz -- Gaussian Fit code served as inspiration. Open source licsense and function from MathWorks: (https://tinyurl.com/y7phn27g)
